# Cocaine-mediated induction of microglial activation involves the ER stress-TLR2 axis

**DOI:** 10.1186/s12974-016-0501-2

**Published:** 2016-02-09

**Authors:** Ke Liao, Minglei Guo, Fang Niu, Lu Yang, Shannon E. Callen, Shilpa Buch

**Affiliations:** Department of Pharmacology and Experimental Neuroscience, 985880 Nebraska Medical Center, University of Nebraska Medical Center, Omaha, NE 68198 USA

**Keywords:** Neuroinflammation, Cocaine, ER stress, Microglial activation, TLR2, ATF4

## Abstract

**Background:**

Neuroinflammation associated with advanced human immunodeficiency virus (HIV)-1 infection is often exacerbated by chronic cocaine abuse. Cocaine exposure has been demonstrated to mediate up-regulation of inflammatory mediators in in vitro cultures of microglia. The molecular mechanisms involved in this process, however, remain poorly understood. In this study, we sought to explore the underlying signaling pathways involved in cocaine-mediated activation of microglial cells.

**Methods:**

BV2 microglial cells were exposed to cocaine and assessed for toll-like receptor (TLR2) expression by quantitative polymerase chain reaction (qPCR), western blot, flow cytometry, and immunofluorescence staining. The mRNA and protein levels of cytokines (TNFα, IL-6, MCP-1) were detected by qPCR and ELISA, respectively; level of reactive oxygen species (ROS) production was examined by the Image-iT LIVE Green ROS detection kit; activation of endoplasmic reticulum (ER)-stress pathways were detected by western blot. Chromatin immunoprecipitation (ChIP) assay was employed to discern the binding of activating transcription factor 4 (ATF4) with the TLR2 promoter. Immunoprecipitation followed by western blotting with tyrosine antibody was used to determine phosphorylation of TLR2. Cocaine-mediated up-regulation of TLR2 expression and microglial activation was validated in cocaine-injected mice.

**Results:**

Exposure of microglial cells to cocaine resulted in increased expression of TLR2 with a concomitant induction of microglial activation. Furthermore, this effect was mediated by NADPH oxidase-mediated rapid accumulation of ROS with downstream activation of the ER-stress pathways as evidenced by the fact that cocaine exposure led to up-regulation of pPERK/peIF2α/ATF4 and TLR2. The novel role of ATF4 in the regulation of TLR2 expression was confirmed using genetic and pharmacological approaches.

**Conclusions:**

xThe current study demonstrates that cocaine-mediated activation of microglia involves up-regulation of TLR2 through the ROS-ER stress-ATF4-TLR2 axis. Understanding the mechanism(s) involved in cocaine-mediated up-regulation of ROS-ER stress/TLR2 expression and microglial activation could have implications for the development of potential therapeutic targets aimed at resolving neuroinflammation in cocaine abusers.

**Electronic supplementary material:**

The online version of this article (doi:10.1186/s12974-016-0501-2) contains supplementary material, which is available to authorized users.

## Background

Cocaine is one of most commonly used illicit drugs in the USA. More than 34 million Americans (16.2 %) aged 15 or older have used cocaine at least once in their lifetime [1]. A variety of disorders of the central nervous system (CNS) have been linked to chronic cocaine abuse including an increased risk of stroke and seizures, cognitive impairment, depression, and, in extreme cases, death [2]. Data from our group has shown that cocaine exposure induces the expression of chemokines and adhesion molecules by binding to its cognate receptor (σ-1R) expressed on a variety of cell types. This in turn may lead to an increased incidence and accelerated progression of HIV-associated neurocognitive disorders (HAND) [3].

Toll-like receptors (TLRs) are well known as a major family of pattern recognition receptors that play a critical role in innate host defense as well as in initiation of adaptive immune responses [4–6]. Recent evidence reveals the expression of TLRs in the CNS where they exert many immune and non-immune functions [7]. More specifically, toll-like receptor-2 (TLR2) is widely expressed in the CNS, and a previous study demonstrated that morphine treatment resulted in up-regulation of TLR2 expression levels in primary microglial cells with a concomitant induction of pro-inflammatory cytokines [8]. However, there are no studies clearly delineating the role of TLR2 in cocaine-induced glial activation.

The endoplasmic reticulum (ER) is well known to play a crucial role in multiple cellular functions such as protein folding, maintenance of Ca^2+^ balance, and cholesterol synthesis [9–11]. Both genetic and environmental insults can perturb the function of the ER and contribute to the development of ER stress. There are three primary stress sensors found in the ER: (1) inositol-requiring kinase 1 (IRE1), (2) protein kinase RNA-like endoplasmic reticulum kinase (PERK), and (3) activating transcription factor 6 (ATF6). When there is an imbalance between protein synthesis and folding capacity in the ER (ER stress), these sensors recognize the misfolded proteins in the lumen of the ER and trigger the unfolded protein response (UPR). Recent studies have revealed that ER stress is involved in the induction of inflammation by triggering signaling pathways to elicit an inflammatory response [12–14]_._ Furthermore, TLR signaling pathways have been shown to cross-talk with ER-stress [15–17].

Microglial cells are the main immune effector cells residing in the CNS and keep the brain environment under constant surveillance. Microglial activation is one of the hallmark features of HAND. In addition, the number of activated microglia is significantly increased among cocaine users [18]. This suggests that microglial activation could be a critical player in cocaine-induced neuroinflammation leading to CNS pathology. The underlying mechanism(s) of microglial activation in the presence of cocaine, however, remain unclear. The present study was aimed to elucidate the molecular mechanism(s) involved in cocaine-mediated microglial activation with a focus on the role of TLR2 and ER stress mediators. This study not only provides a novel mechanism of cocaine-induced microglial activation, but also sheds light on the implications for development of potential therapeutic targets aimed at mitigating neuroinflammation in cocaine abusers.

## Methods

### Reagents

Cocaine hydrochloride (Cat# C5776), apocynin (Cat# A10809), and phenyl-*N*-t-butyl nitrone (PBN) (Cat# B7263) were purchased from Sigma-Aldrich (St Louis, MO, USA). Antibodies purchased from Santa Cruz Biotechnology (Dallas, TX, USA) include TLR2 (Cat# sc-10739), pPERK (Thr 981) (Cat# sc-32577), PERK (Cat# sc-13073), goat anti-rabbit (Cat# sc-2004), and goat anti-mouse (Cat# sc-2005). Antibodies including eukaryotic initiation factor 2α (eIF2α) (Cat# 5324S), peIF2α (Ser51) (Cat# 3398S), and histone H3 (Cat# 9715S) were purchased from Cell Signaling Technology (Danvers, MA, USA). Antibodies including ATF4 (Cat# ab23760) and NF-κB p65 (Cat# ab16502) were purchased from Abcam (Cambridge, MA, USA). Short interfering RNA (siRNA) of TLR2, ATF4, and myeloid differentiation protein 88 (MyD88) were purchased from Thermo Scientific (Hudson, NH, USA).

### Animals

C57BL/6N male mice were purchased from Charles River Laboratories (Wilmington, MA, USA). All of animals were housed under conditions of constant temperature and humidity on a 12-h light, 12-h dark cycle, with lights on at 7:00 am. Food and water were available ad libitum. All animal procedures were performed according to the protocols approved by the Institutional Animal Care and Use Committee of the University of Nebraska Medical Center and the National Institute of Health. Animals were divided into two groups (*n* = 6): (1) saline and (2) cocaine. Cocaine was injected at a dose of 20 mg/kg intraperitoneally for 7 days. On the seventh day, 1 h after the last cocaine injection, the mice were sacrificed, brains removed, and striatal homogenates assessed for levels of TLR2. Mice injected similarly with saline served as controls.

### Primary mouse microglial cell isolation

Primary mouse microglial cell isolation was performed as described previously [19, 20]. Primary microglia cells were obtained from 1- to 3-day-old C57BL/6 newborn pups. After digestion and dissociation of the dissected brain cortices in Hanks buffered salt solution (HBSS, Invitrogen, 14025076) supplemented with 0.25 % trypsin (Invitrogen, 25300-054), mixed glial cultures were prepared by resuspending the cell suspension in Dulbecco’s modified Eagle’s medium (DMEM) (Invitrogen, 11995-065) supplemented with 10 % heat-inactivated fetal bovine serum (FBS, Invitrogen, 16000-044), 100 U/ml penicillin, and 0.1 mg/ml streptomycin. Cells were plated at a density of 20 × 10^6^ cells/flask in 75 cm^2^ cell culture flasks. Cell medium was replaced every 5 days, and after the first medium change, macrophage colony stimulating factor (Invitrogen, PHC9504) at a concentration of 0.25 ng/ml was added to the flasks to promote microglial proliferation. When confluent (7 to 10 days), mixed glial cultures were subjected to shaking at 37 °C at 220 rpm for 2 h, to promote microglia detachment from the flasks. The cell medium, containing the released microglia cells, was collected from each flask and centrifuged at 1000 g for 5 min to collect the cells which were then plated on cell culture plates for all subsequent experiments.

### Mouse microglia isolation

Animals were divided into two groups (*n* = 6/group): (1) saline or (2) cocaine injections. Cocaine was injected at a dose of 20 mg/kg intraperitoneally for 7 days. On the seventh day, 1 h after the last cocaine injection, microglia were isolated from whole brain homogenates by Percoll gradient centrifugation according to previously published reports [19] with slight modifications. Briefly, the brains were homogenized in phosphate-buffered saline (PBS) (pH 7.4) by passing through a 70-μm nylon cell strainer. The homogenates were then centrifuged at 600 g for 6 min. Supernatants were removed, and cell pellets were resuspended in 70 % isotonic Percoll (GE Healthcare, Uppsala, Sweden) at room temperature. A discontinuous Percoll density gradient was layered as follows: 70, 50, 35, and 0 % isotonic Percoll. The gradient was centrifuged for 20 min at 2000*g*, and microglia were collected from the interphase between the 70 and 50 % Percoll layers. Cells were washed and then resuspended in sterile PBS followed by flow cytometry analysis by gating the myeloid cells for the CD11b^+^/CD45dim population.

### BV-2 cell culture

The BV-2 immortalized cell line was obtained from Dr. Sanjay Maggirwar (University of Rochester Medical Center, Rochester, NY, USA) and was grown and routinely maintained in DMEM with 10 % FBS at 37 °C and 5 % CO_2_ and used up to passage 20.

### siRNA transfection

BV-2 cells were seeded into six-well plates and grown to 80 % confluency. The next day, individual targeted siRNA and non-sense siRNA (si-Con) (30 pmol) were mixed with lipofectamine 2000 (2 μl) in 100 μl OptiMEM (Life technologies, 31985062). After 30 min incubation at room temperature, mixed liquids were dropped into cell culture medium (serum free) and incubated for 4 h. Next, the medium was changed to 10 % FBS-containing medium for 20 h incubation. The transfected cells were then ready for use in experiments.

### ROS detection

The Image-iT™ LIVE Green Reactive Oxygen Species (ROS) Detection Kit obtained from Invitrogen (cat# 136007) was used to estimate ROS in live BV2 cells. This experiment was performed according to the manufacturer’s (Life technologies, D-339) recommended protocol. Basically, cells were seeded onto cover slips in 24-well plates 1 day before the experiment. The cells were then washed with HBSS, supplemented with 25 μM carboxy-H2DCFDA working solution, and incubated for 30 min at 37 °C. Subsequently, the cells were washed again with HBSS, and the change in fluorescence was measured using a spectrofluorometer set at 485-nm excitation and 530-nm emission.

### Immunoprecipitation

Immunoprecipitation was performed as described previously [21, 22]. BV2 cells were treated with cocaine (10 μM) for 1 h and then lysed using the Mammalian Cell Lysis kit (Sigma-Aldrich). For each sample, 600 μg of protein was used for immunoprecipitation. Cell lysates were incubated with TLR2 antibody overnight at 4 °C followed by incubation with 30 μl of protein A/G beads (Santa Cruz, 2003) for 1.5 h at 4 °C. The mixture was then centrifuged at 12,000 rpm for 1 min, and the cell pellets were rinsed twice with the lysis buffer (1.0 % NP-40, 0.5 % sodium deoxycholate, 0.1 % SDS, 150 mM NaCl, 9.1 mM Na_2_HPO_4_, 1.7 mM NaH_2_PO_4_) containing proteinase and phosphatase inhibitors. Finally, 30 μl of 2 × western blot loading buffer was added and boiled for 5 min. Then, the protein complexes were detected using 4G10 antibody (Millipore, Cat# ab5320). Input protein (without antibody addition) served as a control to demonstrate that equal amount of total protein was used.

### Western blotting

Treated cells were lysed using the Mammalian Cell Lysis kit (Sigma-Aldrich). Equal amounts of protein were electrophoresed in a sodium dodecyl sulfate-polyacrylamide gel under reducing conditions followed by transfer to PVDF membranes (Millipore, IPVH00010). The blots were blocked with 5 % nonfat dry milk in PBS (137 mM NaCl; 2.7 mM KCl; 10 mM Na2HPO4; 2 mM KH2PO). The western blots were then probed with respective antibodies. The protein amounts loaded were normalized according to the β-actin signal using Mouse Anti-β-Actin antibody (Sigma-Aldrich). The secondary antibodies were HRP conjugated to goat anti-mouse/rabbit IgG (Santa Cruz, sc-2005 and sc-2004).

### Immunocytochemistry

For immunocytochemistry, BV-2 cells were plated on coverslips treated with cocaine (10 μM) for 12 h. The next day, cells were fixed with 4 % paraformaldehyde for 15 min at room temperature followed by permeabilization with 0.3 % Triton X-100 (Fisher Scientific, BP151-1) in PBS. Cells were then incubated with a blocking buffer containing 10 % normal goat serum (NGS) in PBS for 1 h at room temperature followed by addition of rabbit anti-TLR2 (1:200) antibody and incubated overnight at 4 °C. Finally, the secondary Alexa Fluor 594 goat anti-rabbit IgG (Invitrogen, Cat# A11008) was added at a 1:500 dilution for 2 h to detect TLR2. After a final washing with PBS, the coverslips were mounted with the mounting medium (Prolong Gold Anti-fade Reagent; Invitrogen). Fluorescent images were acquired at RT on a Zeiss Observer Z1 inverted microscope. Images were processed using the AxioVs 40 Version 4.8.0.0 software (Carl Zeiss MicroImaging GmbH).

### Immunohistochemistry

Male C57BL/N mice (25 to 30 g) were randomly separated into two groups (*n* = 6/group). One group was administered cocaine (20 mg/kg, IP) daily for 7 days and sacrificed 1 h after the final injection. Mice similarly treated with 0.9 % saline of the same volume served as controls. Animals were transcardially perfused with the fixative, and immunohistochemical procedures were performed as described below. Floating tissue sections (30-μM-thick) were co-incubated with primary anti-mouse ionized calcium-binding adapter molecule 1 (Iba1) (Abcam, Cat# ab15690), anti-rabbit TLR2, anti-goat Iba1 (Abcam, Cat# ab5076), and anti-mouse CD68 antibody (Dako, Cat# M0814) overnight at 4 °C. Alexa Fluor 488 conjugated anti-mouse or anti-goat (Life Technologies, Cat# A11001; Invitrogen, Cat# A11055) and Alexa Fluor 594 goat anti-rabbit secondary antibodies (Invitrogen, Cat# A11008) were added for 2 h to detect Iba1 and TLR2 followed by mounting of sections with DAPI (Invitrogen, 36935). Fluorescent images were acquired at room temperature on a Zeiss Observer Z1 inverted microscope (Carl Zeiss, German); images were processed using AxioVs 40 4.8.0.0 software (Carl Zeiss MicroImaging). Photographs were acquired using an AxioCam MRm digital camera (Carl Zeiss, German).

### RNA extraction, reverse transcription, and quantitative polymerase chain reaction (qPCR)

Total RNA was extracted using Trizol reagent (Invitrogen, 15596-018). Briefly, monolayer cells in six-well plates were washed with PBS and lysed directly adding 1 ml Trizol. Cell lysate was aspirated into new 1.5 ml microcentrifuge tubes followed by addition of 0.2 ml of chloroform. After extensive mixing, the samples were centrifuged at 12,000*g* for 15 min at 4 °C. The upper aqueous phase was transferred to a new tube followed by addition of 500 μl of isopropyl alcohol. Samples were incubated for 10 min and centrifuged again to precipitate total RNA. Total RNA was dissolved in DEPC-treated H_2_O and quantified. Reverse transcription reactions were performed using a Verso cDNA kit (Invitrogen, AB1453/B). The Reaction system (20 μl) included 4 μl 5 × cDNA synthesis buffer, 2 μl dNTP mix, 1 μl RNA primer, 1 μl RT enhancer, 1 μl Verso enzyme Mix (Invitrogen, AB-1453/B), 1 μg total RNA template, and a variable volume of water. Reaction conditions were set at 42 °C for 30 min. The qPCRs were performed by using SYBR Green ROX qPCR Mastermix (Qiagen, 330510). Reaction systems were set up as follows: 10 μl SYBR Green Mastermix, 0.5 μl forward primers, 0.5 μl reverse primers, and 9 μl DEPC-treated H_2_O. Ninety-six-well plates were placed into a 7500 fast real-time PCR system (Applied Biosystems, Grand Island, NY). Mouse primers for TNFα, IL-6, and MCP-1 were purchased from (Invitrogen, Mm00443258, Mm00446190, and Mm00441242).

### TNFα and MCP-1 analyses by ELISA

Supernatant fractions collected from BV-2 cells that were treated with cocaine in the presence or absence of the indicated inhibitors or siRNA were examined for secreted TNFα and MCP-1 protein levels using the commercially available ELISA kits (R&D Systems, MTA00B and MJE00). The data presented represent results obtained from three independent experiments.

### Flow cytometry

Cells were stained for flow cytometry according to a previously published protocol, with some modifications [23]. After detaching from plates, BV-2 cells were washed once and resuspended in 1 ml of staining buffer (PBS with 2 % FBS). Cells were counted and incubated with anti-CD16/CD32 (1 μg/10^6^ cells) to block FcγII/III receptors. TLR2 antibody [T2.5] (FITC) (Abcam, Cat# ab59711) was added to the cells, and the mixtures were incubated for 10 min on ice in the dark. Cell suspensions were then exposed to direct fluorescent light for 15 min at room temperature. Following two washes with staining buffer, cells were fixed with 0.5 % PFA. The cells were analyzed on an LSR II flow cytometer (BD Biosciences, San Jose, CA, USA) using FACSDiva software.

### Chromatin immunoprecipitation (ChIP) assay

The ChIP assay was performed according to the manufacturer’s instructions (Upstate, Billerica, MA, USA) with slight modifications. After treatment of the cells, 18.5 % fresh formaldehyde was added directly into the medium at a final concentration of 1 % formaldehyde and incubated for 10 min at room temperature followed by quenching with 125 mM glycine. The cells were then detached using 2 ml of pre-chilled PBS containing 1 × protease inhibitor mixture. The cell pellet was harvested by spinning at 800*g* at 4 °C, and lysis buffer was added (provided in the kit) to harvest the nuclei. DNA was then sheared by sonication. A total of 50 μl of the sheared cross-linked chromatin was then mixed with 20 ml protein A magnetic beads and 5 mg of immunoprecipitating Abs against ATF4, acetyl histone H3 (as a positive control), and normal rabbit IgG (as a negative control) diluted in 450 ml dilution buffer overnight at 4 °C. The magnetic beads binding the Ab-chromatin complex were then washed with 0.5 ml each of a series of cold wash buffers in the order of low salt buffer, high salt buffer, LiCl buffer, and Tris-EDTA buffer. The crosslinking of protein-DNA complexes were reversed to free DNA by incubation at 62 °C for 2 h and purified using DNA purification spin columns following the manufacturer’s instructions. Finally, the purified DNA was amplified (35 cycles) via PCR to identify the promoter region containing ATF4 binding site “CGGTGACGCTGTCC.” The sequences of the primers used to identify the TLR2 promoter bound to ATF4 were as follows: sense, TGTGTCCGCAATCATAGT and antisense, CGCTTTGTCTGAGGTTTC.

### Statistical analysis

Statistical analysis was performed using one-way analysis of variance with a post hoc Student *t* test. Results were judged statistically significant if *p* < 0.05 by analysis of variance.

## Results

### Cocaine-induced up-regulation of TLR2 expression in mouse microglia

**Fig. 1:**
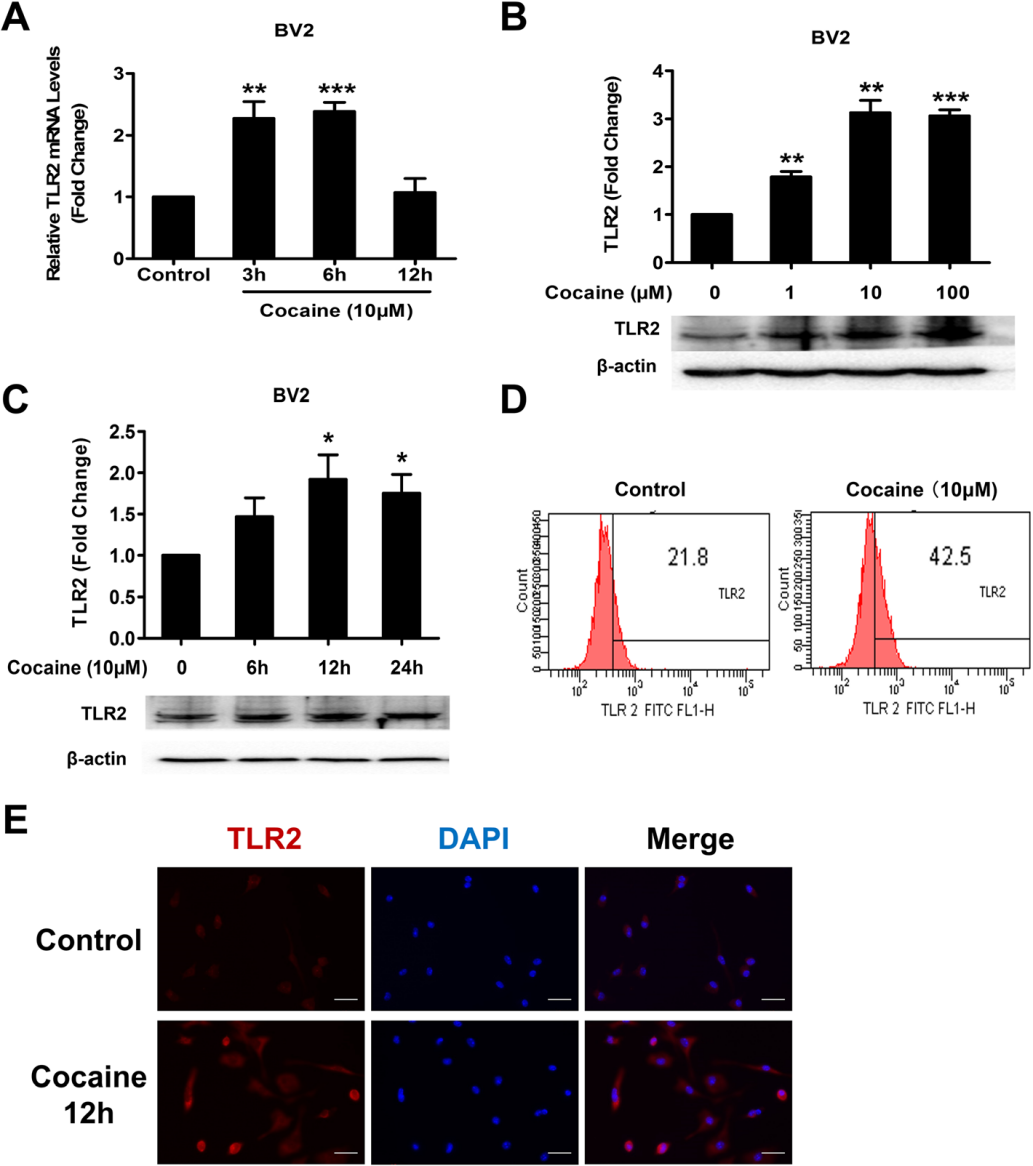
Cocaine-mediated up-regulation of TLR2 expression in mouse microglial cells. **a** Time-course of cocaine-mediated induction of TLR2 expression in BV2 cells by qPCR. **b** Dose curve of cocaine-mediated induction of TLR2 expression in BV2 cells. Cells were treated with various conc. of cocaine (1–100 μM). **c** Time-course of cocaine-mediated induction of TLR2 expression in BV2 cells. **d** BV2 cells were treated with cocaine (10 μM). TLR2 protein levels on the surface of BV2 cells was analyzed by flow cytometry. A total of 10,000 events were acquired in all the experiments using FACS flow cytometry and analyzed using the BD DIVA software (BD Biosciences). **e** Representative image of TLR2 staining in BV2 cells. All data are presented as mean ± SD of three individual experiments. **p < 0.01, ***p < 0.001 vs control group (Student’s *t* test)

It has been well-documented that cocaine can impair innate immune responses via regulating the immune cell activity and cytokine production [24–27]. Microglial cells, as the key player of immune response in the CNS, are the predominant cell types assessed for cocaine-mediated activation of CNS inflammation. Additionally, TLR2, as the most highly expressed TLR family member in microglia from rodents as well as humans [28–30], has been shown to contribute to activation of microglia. To investigate whether cocaine affects TLR2 expression in mouse microglia, BV2 cells were exposed to 10 μM cocaine for varying times (3, 6, 12 h) and an initial screen was done to identify the RNA levels of TLR2 by qPCR. As shown in Fig. 1a, exposure of BV2 cells to cocaine induced a time-dependent up-regulation of TLR2 with maximal up-regulation (2.4-fold, *p* = 0.00071) observed at 6 h. To confirm whether increased mRNA levels of TLR2 translated into enhanced protein levels, western blot analyses were done to determine the dose- and time-course of cocaine-mediated induction of TLR2 in BV2 cells. For the dose curve, BV2 cells were treated with cocaine at varying concentrations (1, 10, 100 μM) for 12 h, followed by protein extraction and assessment of TLR2 expression by western blot. As shown in Fig. 1b, cocaine up-regulated TLR2 expression in BV2 cells in a concentration-dependent manner with maximal up-regulation at 10 μM cocaine (~3.1-fold, *p* = 0.0011). Based on this, we chose 10 μM of cocaine for all our subsequent studies. It must be pointed out that this concentration of cocaine, while high, is physiologically relevant since the levels of cocaine in the plasma of human cocaine addicts is often found in the range of 0.4–1.6 μM [31] and that in the plasma of tolerant abusers is up to 13 μM [32]. For the time-course study, BV2 cells were exposed to cocaine (10 μM) for varying time points (6, 12, 24 h), and as shown in Fig. 1c, the TLR2 levels were induced in the presence of cocaine from 6 to 24 h with the maximal induction of TLR2 at 12 h (1.9-fold, *p* = 0.036). Additionally, TLR2 protein levels were also assessed by flow cytometric analyses. As shown in Fig. 1d, BV-2 cells treated with cocaine displayed an increased cell surface expression of TLR2 compared with the untreated cells. Confirmation of these findings by immunostaining also revealed increased TLR2 levels in BV2 cells at 12 h following cocaine (10 μM) exposure. Images were captured by fluorescence microscopy using a ×40 objective lens. As shown in Fig. 1e, the intensity of TLR2 fluorescence was dramatically induced by cocaine. Taken together, these findings suggested that cocaine mediated the induction TLR2 at both the mRNA and protein levels in BV2 cells.

### Cocaine-induced TLR2-dependent microglial activation

**Fig. 2:**
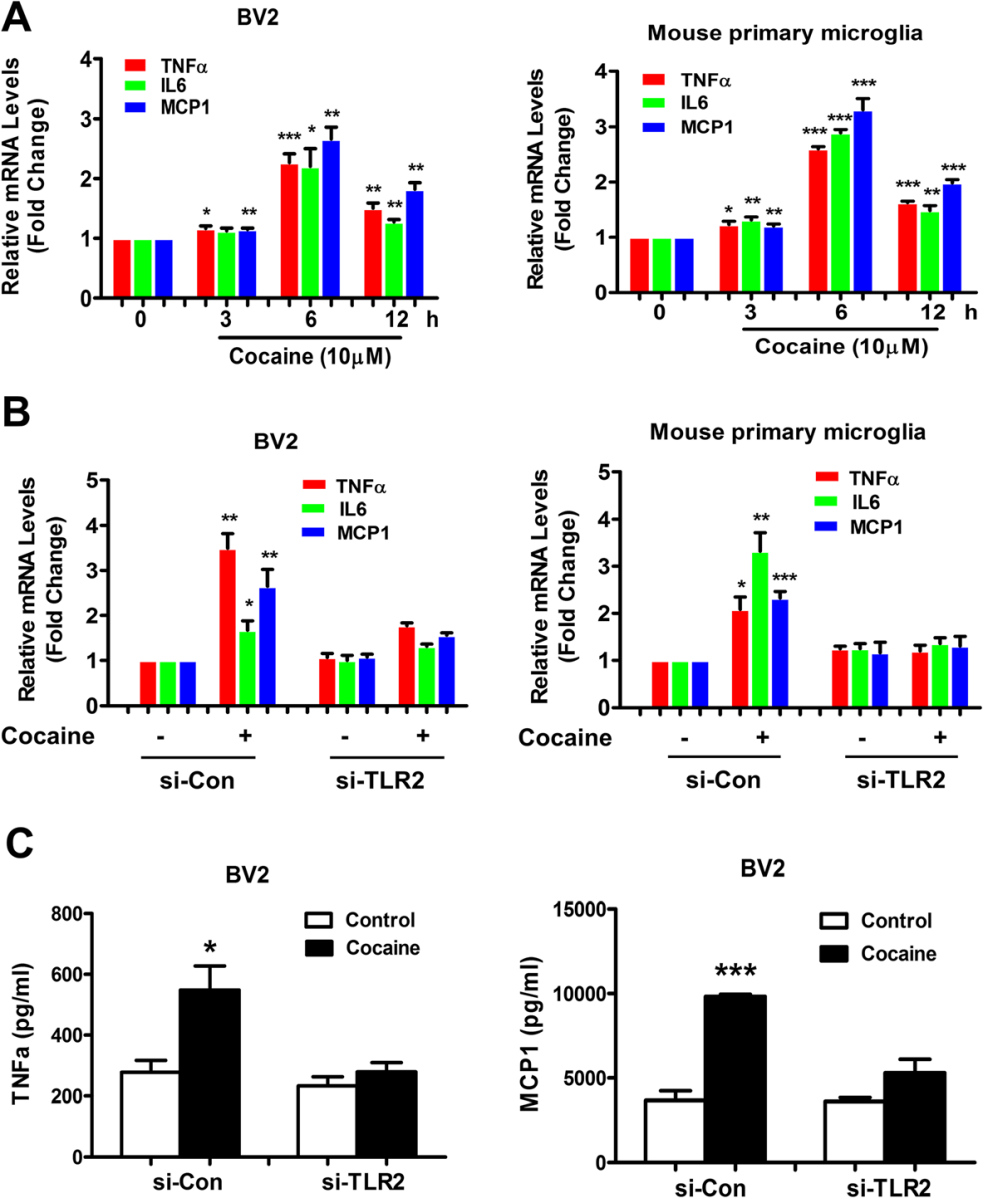
TLR2-dependent cocaine-mediated microglial activation. **a** Time-course of cocaine (10 μM) mediated induction of TNFα, IL-6, and MCP-1 mRNA expression in BV2 and mouse primary microglial cells by qRT-PCR. **b** BV2 and mouse primary microglial cells transfected with TLR2 siRNA but not si-Con resulted in abrogation of cocaine-mediated induction of TNFα, IL-6, and MCP-1 mRNAs by qPCR using 18S rRNA as internal control. **c** ELISA analysis revealed the concentrations of TNFα and MCP-1 proteins in the supernatants derived from BV2 cells knocked down for TLR2. All data are presented as mean ± SD of three individual experiments. *p < 0.05, **p < 0.01, ***p < 0.001 vs control group (Student’s *t* test)

Since TLR2 engagement is known to induce microglial activation [33], we sought to investigate whether cocaine could activate microglia and, if so, if cocaine-mediated microglial activation was regulated through TLR2. In the current study, we examined whether cocaine induced the release of pro-inflammatory cytokines from BV2 cells as an indicator of microglial activation. Firstly, BV-2 cells were treated with 10 μM cocaine at various time points (0–12 h) and assessed for expression of pro-inflammatory cytokine mRNAs. As shown in Fig. 2a, exposure of cells with cocaine induced a time-dependent up-regulated expression of mRNAs specific for pro-inflammatory cytokines including TNFα, IL-6, and MCP-1 using the qPCR assay. TNFα, IL-6, and MCP-1 mRNAs were maximally up-regulated (2.3-fold, *p* = 0.00095; 2.2-fold, *p* = 0.015; 2.7-fold, *p* = 0.0011, respectively) at 6 h compared with the control group. These findings were also validated in primary mouse microglial cells. In the primary cells, expression of TNFα, IL-6, and MCP-1 mRNAs was maximally up-regulated (2.6-fold, *p* = 2.8 × 10^−6^; 2.9-fold, *p* = 6.2 × 10^−6^; 3.3-fold, *p* = 0.00030, respectively) at 6 h compared with the untreated control cells.

The next logical step then was to elucidate whether induction of TLR2 played a role in cocaine-mediated microglial activation. BV-2 cells were transfected with either TLR2 siRNA or si-Con for 24 h followed by exposure of cells to cocaine and assessed for release of pro-inflammatory cytokines in the BV2 cell supernatants. As shown in Fig. 2b, c, cells (BV-2 and mouse primary microglial cells) transfected with the TLR2 siRNA failed to demonstrate cocaine-mediated up-regulation of TNFα, IL-6, and MCP-1 mRNAs. On the other hand, as expected, cells transfected with the si-Con did exhibit cocaine-mediated increase of pro-inflammatory cytokine mRNAs. These findings thus underpinned the role of TLR2 in cocaine-mediated induction of microglial activation. Further corroboration of these findings was done by assessing culture supernatants using ELISA for analyses of the pro-inflammatory cytokines in cells transfected with the respective siRNAs, followed by exposure to cocaine. As shown in Fig. 2c, in cells knocked down for TLR2 expression, cocaine failed to induce secretion of pro- inflammatory cytokines.

### Involvement of ROS in cocaine-mediated up-regulation of TLR2 expression and microglial activation

**Fig. 3:**
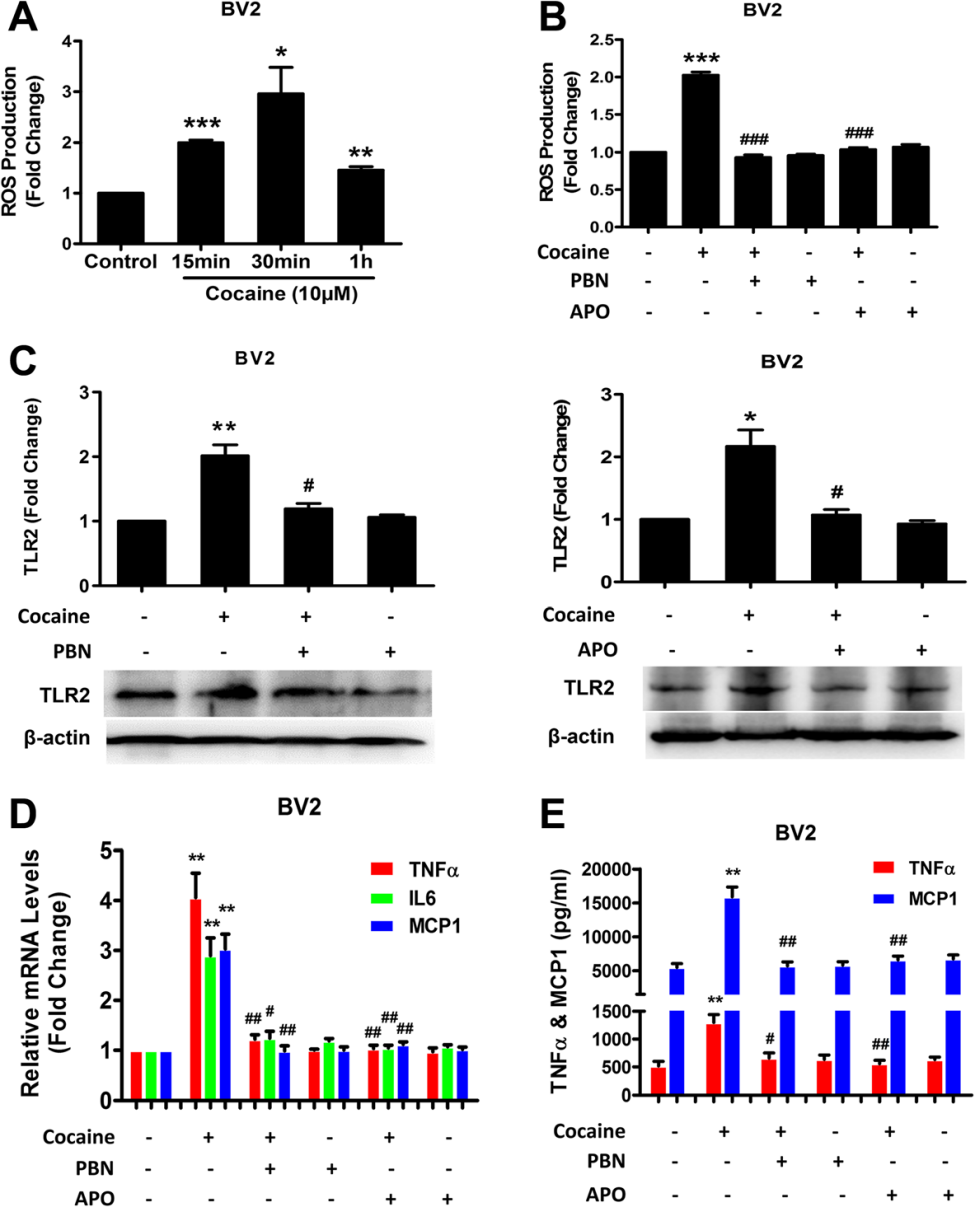
Cocaine-mediated up-regulation of TLR2 protein and microglial activation involves ROS. **a** ROS generation in BV2 cells exposed to cocaine assessed by DCFH-DA. **b** Effects of PBN (ROS scavenger) and APO (NADPH oxidase inhibitor) on cocaine induced ROS formation in BV2 cells. **c** Effects of PBN and APO on cocaine-mediated induction of TLR2 protein. TLR2 protein levels were measured at 12 h after cocaine treatment. For western blot analysis, the induction of TLR2 protein was normalized to β-actin. **d** Effects of PBN and APO on cocaine-mediated expression of TNFα, IL-6, and MCP-1 mRNAs. Relative levels of TNFα, IL-6, and MCP-1 mRNAs were analyzed by qPCR using 18S rRNA as an internal control. **e** Effects of PBN and APO on cocaine-mediated induction of TNFα and MCP-1 protein. ELISA analysis revealed the concentrations of TNFα and MCP-1 proteins in the supernatants of BV2 cells pretreated with PBN and APO followed by exposure to cocaine. All data are presented as mean ± SD of three individual experiments. *p < 0.05, **p < 0.01, ***p < 0.001 vs control group. ^#^ *p* < 0.05, ^##^ *p* < 0.01, ^###^ *p* < 0.001 vs cocaine group (Student’s *t* test)

Having established that cocaine both up-regulated the expression of TLR2 protein levels and also induced microglial activation, we next sought to explore the mechanism(s) underlying these processes. It has been well-documented that cocaine can up-regulate the expression of ROS in CNS cells [34–36]. We thus rationalized that generation of ROS could be involved in cocaine-mediated up-regulation of TLR2 protein and the ensuing microglial activation. Microglial BV2 cells were exposed to cocaine for varied time points (0–1 h) and monitored for ROS production using the DCFH-DA assay. As shown in Fig. 3a, exposure of cells to cocaine resulted in significant ROS production with the peak induction of ROS at 30 min (3.0-fold, *p* = 0.02) post cocaine treatment. Specificity of this effect was examined in cells pretreated with the ROS scavenger phenyl-*N*-t-butyl nitrone (PBN), which significantly inhibited cocaine-mediated induction of ROS generation (Fig. 3b and Additional file 1: Figure S1). Next, we sought to examine whether NADPH oxidase contributed to cocaine-mediated generation of ROS. As shown in Fig. 3b and Additional file 1: Figure S1, pretreatment of cells with the NADPH oxidase inhibitor, apocynin (APO), also resulted in significant inhibition of cocaine-mediated induction of ROS production. The next step was to assess whether cocaine-mediated ROS generation was involved in cocaine-mediated induction of TLR2. For this, BV2 cells were pretreated with the ROS inhibitors (PBN or APO) followed by exposure to cocaine that was subsequently followed by assessment of TLR2 protein by western blot analysis. As shown in Fig. 3c, blocking cocaine-mediated generation of ROS significantly abolished cocaine-mediated induction of TLR2 in BV2 cells. We next examined whether cocaine-mediated generation of ROS production resulted in microglial activation. Similar to the effect seen for TLR2 protein, pretreatment of cells with the ROS inhibitors blocked cocaine-mediated up-regulation of both TNFα and MCP-1 at the RNA and protein levels (Fig. 3d, e).

### Role of oxidative-ER stress in cocaine-mediated up-regulation of both TLR2 protein and microglial activation in BV2 cells

**Fig. 4:**
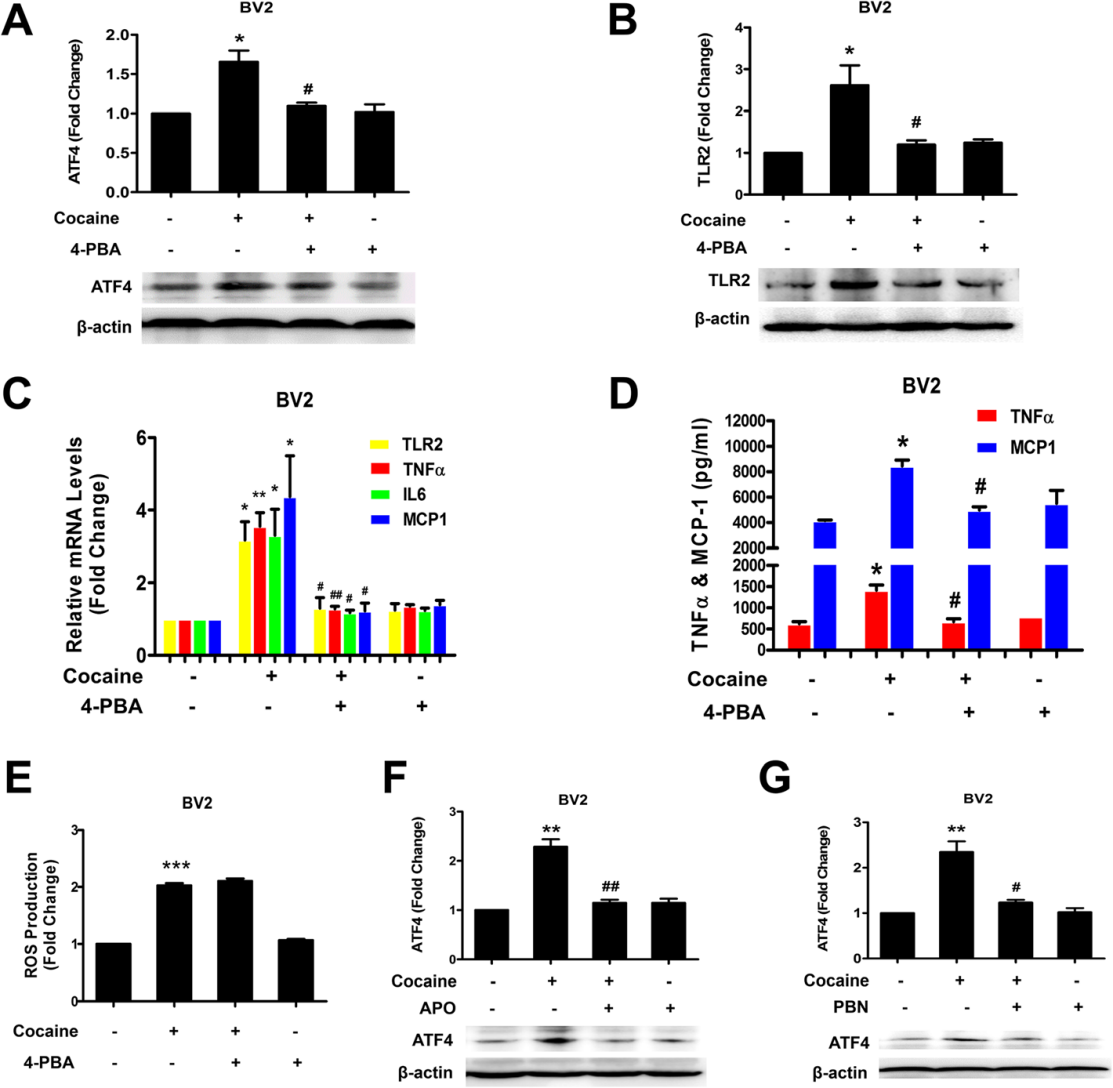
Role of Oxidative-ER stress in cocaine-mediated up-regulation of TLR2 and microglial activation in BV2 cells. **a**, **b** Effect of pharmacological inhibitor of ER-stress 4-PBA on cocaine-mediated up-regulation of ATF4 and TLR2 proteins in BV2 cells. **c** Effect of 4-PBA on cocaine-mediated induction of TLR2, TNFα, IL-6, and MCP-1 mRNAs. Relative levels of TLR2, TNFα, IL-6, and MCP-1 mRNAs were analyzed by qPCR using 18S rRNA as an internal control. **d** Effects of 4-PBA on cocaine-mediated induction of TNFα and MCP-1 proteins assessed by ELISA. **e** 4-PBA did not ameliorate cocaine-mediated induction of ROS generation. **f**, **g** Effects of APO (NADPH oxidase inhibitor) and PBN (ROS scavenger) on cocaine-mediated up-regulation of ATF4 protein in BV2 cells. All data are presented as mean ± SD of three individual experiments. *p < 0.05, **p < 0.01, ***p < 0.001 vs control group. ^#^ *p* < 0.05, ^##^ *p* < 0.01 vs cocaine group (Student’s *t* test)

ER stress regulates inflammatory responses which mediate production of cytokines and chemokines such as IL-6, IL-8, and MCP-1 [37]. Additionally, ER stress greatly enhances the production of beta interferon (IFN-β), IL-6, IL-1β, and IL-23 in response to the bacterial component lipopolysaccharide (LPS), which implies that cross-talking between ER stress and TLR signaling plays an important role in inflammatory responses [38, 39]. We thus rationalized that triggering ER-stress could drive cocaine-mediated up-regulation of TLR2 protein and the ensuing microglial activation. Firstly, we examined the involvement of the ER-stress pathway in cocaine-mediated responses. Exposure of BV2 cells to cocaine resulted in a time-dependent increase in ER-stress pathway PERK/elf2α/ATF4, with activation as early as 15 min following exposure (Additional file 2: Figure S2). Specificity of these signaling pathways was subsequently assessed using a pharmacological approach. Pretreatment of BV2 cells with an ER-stress inhibitor (sodium 4-phenylbutyrate, 4-PBA) for 1 h resulted in abrogation of cocaine-induced ATF4 expression (Fig. 4a). We next sought to examine the functional role of ER-stress in TLR2 expression and microglial activation induced by cocaine. BV2 cells were pretreated with an inhibitor of ER-stress signaling (4-PBA) for 1 h, followed by exposure of BV2 cells to cocaine for 12 h with assessment of TLR2 protein and pro-inflammatory cytokine release. As shown in Fig. 4b, pretreatment of cells with 4-PBA resulted in amelioration of cocaine-mediated induction of TLR2 expression. As regards microglial activation, inhibition of ER-stress significantly reduced cocaine-mediated expression of TNFα, IL-6, and MCP-1 mRNAs as shown by qPCR analysis (Fig. 4c). Amelioration of cocaine-mediated elevation of TNFα, and MCP-1 protein levels in cells pretreated with the ER stress inhibitor was further confirmed by ELISA analysis.

Having determined that cocaine-mediated ER stress played an important role in cocaine-mediated induction of TLR2 protein and microglial activation, we next sought to examine the molecular link between cocaine exposure and ER stress activation. Generation of ROS has been implied in the activation of PERK-dependent ER stress signaling [40, 41]. Herein, we examined whether ROS was also the precedent signal for cocaine-mediated ER pathway activation. BV2 cells were pretreated with 4-PBA for 1 h followed by exposure of cells to cocaine and subsequent assessment of ROS production by DCFH-DA assay. As shown in Fig. 4e, pretreatment with 4-PBA had no effect on cocaine-mediated ROS production implying thereby that ROS production was upstream of ER-stress activation. Furthermore, both the ROS inhibitors (PBN and APO) blocked cocaine-mediated activation of ER-stress as evidenced by the down-regulation of ATF4 levels (Fig. 4f, g). Taken together, our findings suggest that ROS lies upstream of ER signaling and participates in cocaine-mediated microglial activation.

### Role of ATF4 in cocaine-mediated up-regulation of TLR2 expression and microglial activation

**Fig. 5:**
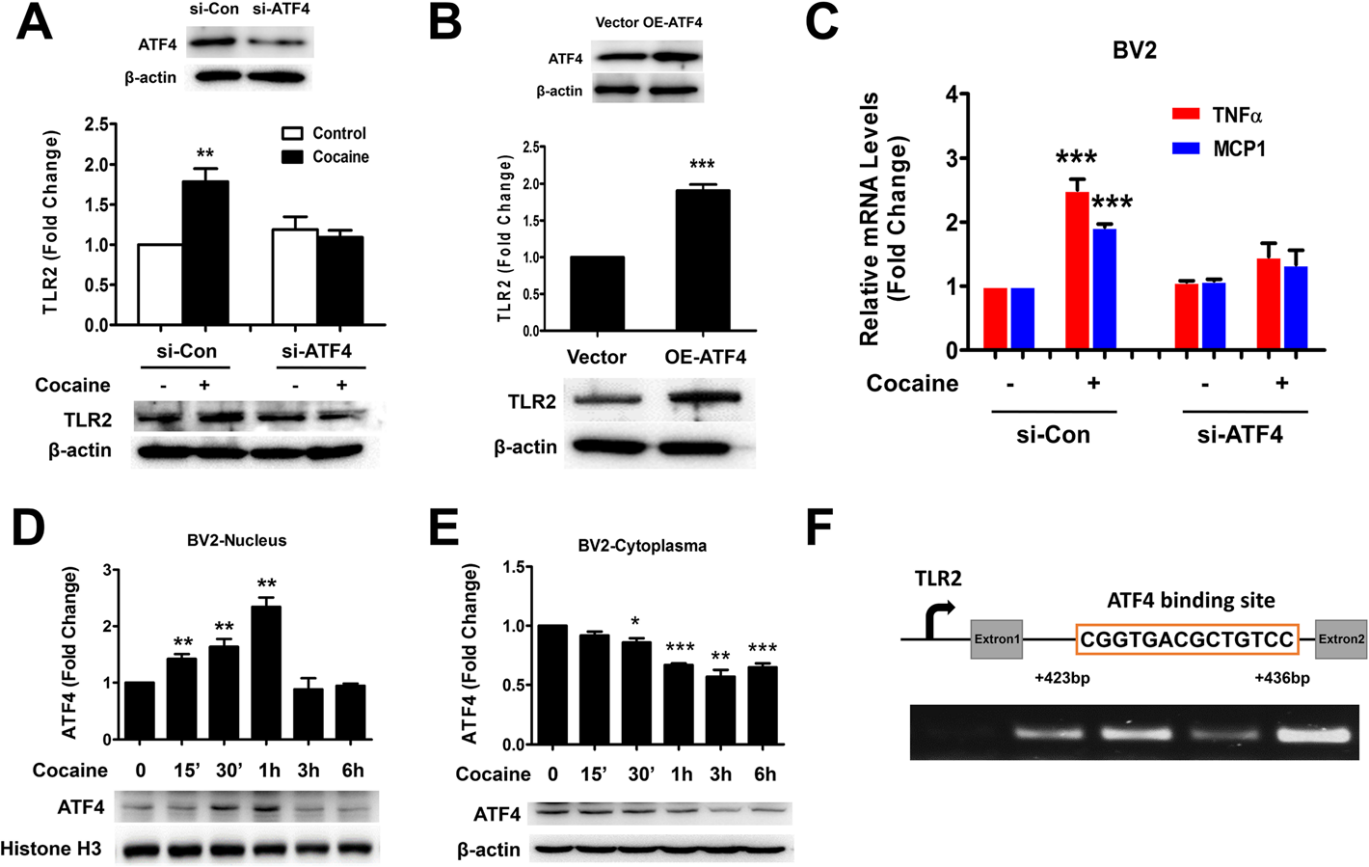
Role of ATF4 in cocaine-mediated up-regulation of TLR2 expression and microglial activation. **a** siRNA transfection was used to knock down ATF4. ATF4 siRNA but not si-Con inhibited cocaine-mediated induction of TLR2 protein. **b** Overexpression of ATF4 up-regulated TLR2 protein levels. **c** Role of ATF4 in cocaine-mediated induction of TNFα and MCP-1 mRNAs. **d**, **e** Cocaine-mediated nuclear translocation of ATF4 in BV2 cells. **f** Schematic illustration of ATF4 binding sequence on the promoter region of TLR2. ChIP assay demonstrating cocaine-mediated binding of ATF4 to the TLR2 promoter. All data are presented as mean ± SD of three individual experiments. *p < 0.05, **p < 0.01, ***p < 0.001 vs control group (Student’s *t* test)

Having established that blocking ROS production and ER-stress abolished cocaine-mediated up-regulation of ATF4, TLR2 protein and microglial activation, we next rationalized that ATF4 was critical for cocaine-mediated up-regulation of TLR2 as well as for microglial activation. To elucidate the role of ATF4, BV2 cells were transfected with either ATF4 siRNA or si-Con followed by exposure of cells to cocaine for 12 h and assessed subsequently for TLR2 levels. As shown in Fig. 5a, knocking down ATF4 significantly blocked cocaine-mediated up-regulation of TLR2 protein. Reciprocally, Fig. 5b showed that overexpression of ATF4 resulted in significant (1.9-fold, *p* = 0.00042) up-regulation of cocaine-mediated induction of TLR2. Similarly, the role of ATF4 was also examined in cocaine-mediated activation of microglia. As shown in Fig. 5c, cocaine-mediated induction of TNFα and MCP-1 expression was attenuated in cells transfected with ATF4 siRNA but not in cells transfected with si-Con. These findings underscored the role of ATF4 in cocaine-mediated induction of microglial activation.

We next sought to enquire whether cocaine could also mediate nuclear translocation of ATF4 resulting ultimately in increased transcription of TLR2. BV2 cells were exposed to cocaine for varying time points (0 to 6 h) followed by assessment of ATF4 translocation in the nuclear fractions. As shown in Fig. 5d, e, exposure of BV2 cells to cocaine resulted in a time-dependent increase in translocation of ATF4 into the nucleus with a maximal response at 1 h (2.3-fold, *p* = 0.0013) and a concomitant decrease in the cytoplasm.

It is well-recognized that nuclear translocation of transcription factors is necessary for accessing and binding to the promoter region of a gene. Intriguingly, using the TFSEARCH software we found a predicted ATF4 binding site in the intron of the gene instead of its presence at a traditional promoter site upstream of the transcription start site. This led to a speculation that ATF4 could be binding to the intronic promoter of the TLR2 gene. Adding credence to this hypothesis are reports identifying an intronic promoter in the murine proteinase 3 gene [42] and another report that described the binding of ATF4 to an intronic promoter leading, in turn, to regulated expression of Siah2 mRNA in response to ER stress [43]. Interestingly, ATF4-binding site is also present in the first intron of the human VEGFA gene [44]. To assess whether cocaine mediated the binding of ATF4 to the intronic region of TLR2, we performed the ATF4 ChIP assay. BV2 cells were treated with cocaine for 1 h followed by RNA extraction and processed using a ChIP assay kit. As shown in Fig. 5f, exposure of BV2 cells to cocaine resulted in enhanced binding of ATF4 to the TLR2 intronic promoter.

### TLR2 phosphorylation, NF-ĸB translocation, and MyD88 are involved in cocaine-mediated microglial activation

**Fig. 6:**
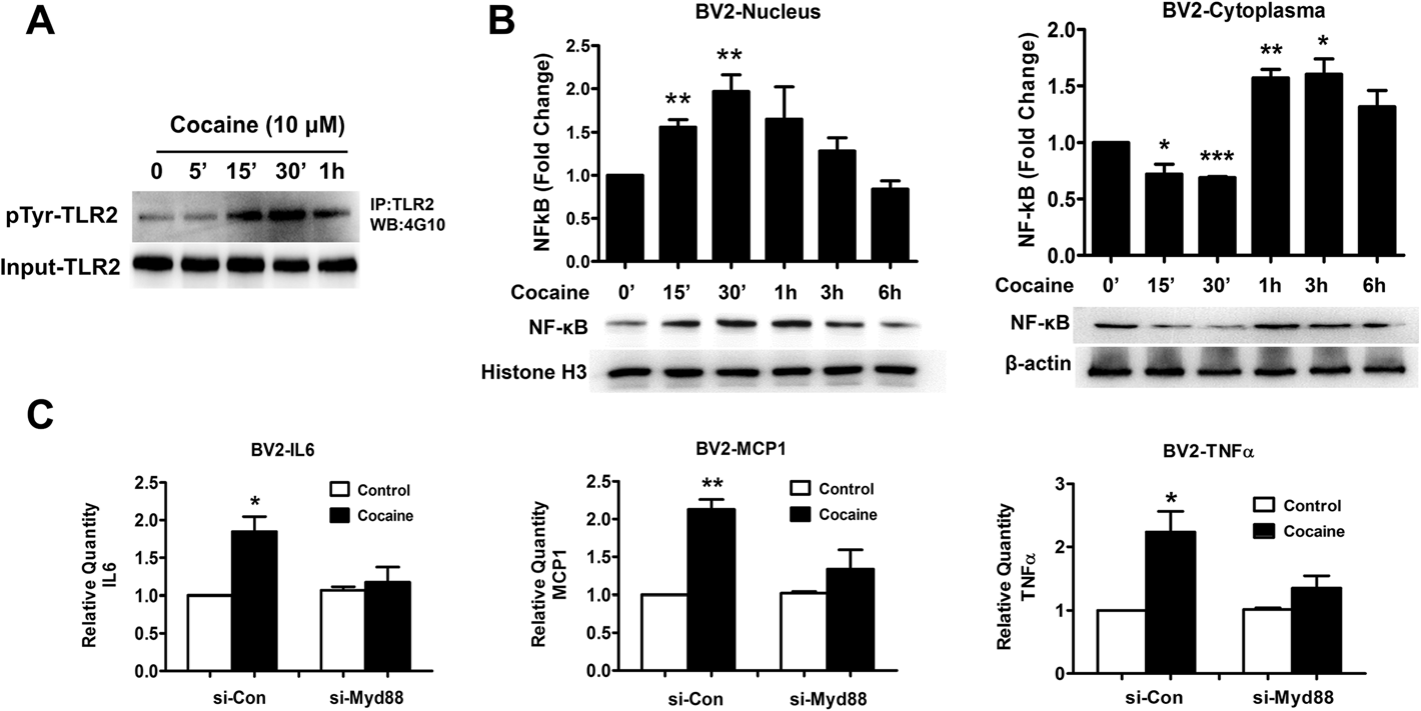
Cocaine-mediated induction of phosphorylation-dependent TLR2 signaling pathway and microglial activation. **a** Cocaine-mediated induction of phosphorylation of TLR2 in BV2 cells. **b** Cocaine-mediated induction of NF-κB nuclear translocation in BV2 cells. **c** siRNA transfection was used to knock down Myd88. Myd88 siRNA but not si-Con inhibited cocaine-mediated induction of IL-6, MCP-1, and TNFα mRNAs, analyzed by qPCR using 18S rRNA as internal control. All data are presented as mean ± SD of three individual experiments. *p < 0.05, **p < 0.01, ***p < 0.01 vs control group (Student’s *t* test)

Having demonstrated that cocaine mediated up-regulation of TLR2 protein and microglial activation, we next sought to examine whether cocaine could also activate the TLR2 signaling pathway(s) leading, in turn, to downstream activation of microglia. To explore the possibility that cocaine exposure could also increase phosphorylation of TLR2, lysates from cocaine-exposed BV2 cells were immunoprecipitated with TLR2 antibody and subsequently assessed for TLR2 tyrosine phosphorylation using the specific tyrosine antibody 4G10. As shown in Fig. 6a, cocaine-induced phosphorylation of TLR2 was observed as early as 15 min after cocaine exposure with a peak at 30 min. Next, we sought to investigate whether cocaine could also promote nuclear translocation of NF-ĸB. For this, following exposure of BV2 cells to cocaine, cytosolic and nuclear protein extracts were monitored for levels of NF-ĸB at the indicated time points. As shown in Fig. 6b, as early as 15 min posttreatment, there was increased expression of NF-ĸB in the nucleus with a peak expression at 30 min (2.0-fold, *p* = 0.0075) and a concomitant decrease in cytosolic NF-ĸB levels. To further discern the role of MyD88 in cocaine-mediated microglial activation, BV2 cells were first transfected with either MyD88 or si-Con followed by exposure of cells to cocaine for 6 h and assessment of cellular lysates for expression of pro-inflammatory cytokine mRNAs. As shown in Fig. 6c, knockdown of MyD88 resulted in reversal of cocaine-mediated induction of TNFα, IL-6, and MCP-1 mRNA. Taken together, our findings thus underpin the role of cocaine in mediating microglial activation via TLR2 phosphorylation, MyD88 activation, and NF-ĸB translocation.

### Cocaine up-regulated the expression of TLR2 and microglial activation in mouse microglia in vivo

**Fig. 7:**
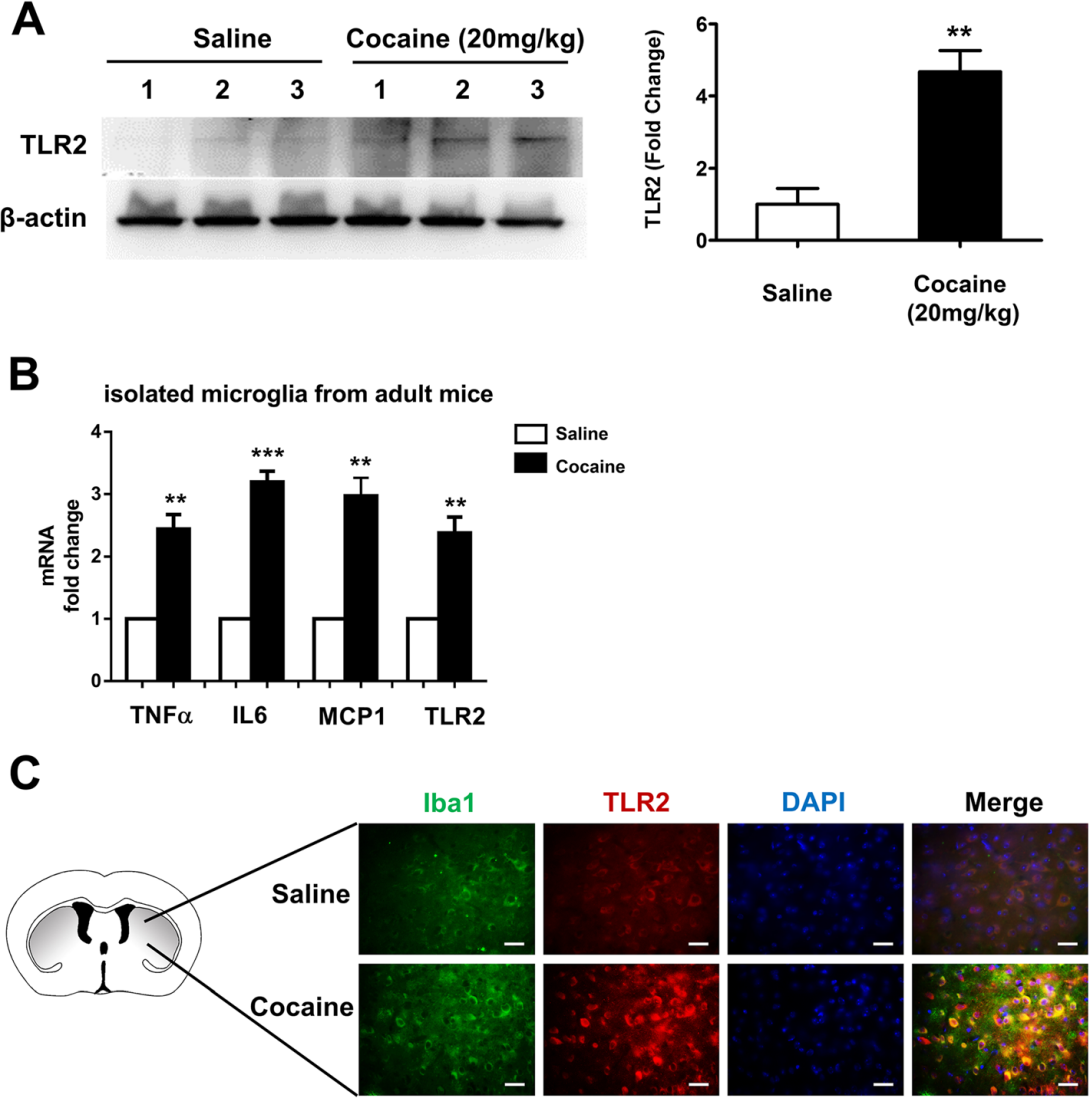
Cocaine-mediated up-regulation of both TLR2 and microglial activation in mouse microglia in vivo. **a** Chronic cocaine administration to mice resulted in significant induction of TLR2 protein in the striatum of mice compared with saline-injected controls. **b** Effect of cocaine on the expression of TLR2 and microglial activation in microglia isolated from the brains of cocaine/saline administered mice. Total RNA isolated from microglia was subjected to qPCR analyses using primer sets specific for TNFα, IL-6, MCP-1, and TLR2. **c** Floating tissue sections were double immunostained with antibodies specific for Iba1/TLR2 and assessed for co-localization of Iba1/TLR2 positive cells using the Nuance multi spectrum imaging system. Cocaine exposure resulted in increased numbers of TLR2 (*red*) positive microglia (Iba1, *green*) in the mouse brain. All data are presented as mean ± SD of three individual experiments. **p < 0.01, ***p < 0.01 vs saline group (Student’s *t* test)

After ascertaining the role of cocaine in mediating induction of TLR2 expression and microglial activation in vitro, we next sought to determine whether cocaine could also induce TLR2 protein and microglial activation in vivo. Groups of mice (*n* = 6) were treated with cocaine once a day (20 mg/kg, IP) for seven consecutive days, and 1 h following the last cocaine injection, mice were sacrificed, brains removed, and striatal homogenates assessed for levels of TLR2 protein. Mice injected similarly with saline served as controls. As shown in Fig. 7a, our findings demonstrated that chronic cocaine administration to mice resulted in significant induction of TLR2 protein in the striatum compared with saline-injected controls (4.7-fold, *p* = 0.0077). In addition, following seven days of cocaine administration, we also isolated microglia from the brains of cocaine/saline administered mice by Percoll gradient method and sorting cells by flow cytometry for CD11b+/CD45dim population. Total RNA isolated from the microglia was subjected to qPCR analyses. As shown in Fig. 7b, cocaine exposure resulted in up-regulation of IL-6, TNFα, MCP-1, and TLR2 mRNAs in isolated adult mice microglial cells. Taken together, our findings thus established that cocaine exposure resulted in up-regulation of TLR2 expression and microglial activation in vivo.

To further examine whether following cocaine exposure microglial cells in vivo contributed to increased TLR2 expression as well as increased microglial activation, brain sections from cocaine- and saline-administered mice were double immunostained with the microglial marker Iba1 and TLR2 and Iba1 and microglial activation marker CD68. As shown in Fig. 7c, in the presence of cocaine, there was increased co-localization of Iba1-positive microglia (green) with TLR2 (red). Additionally, more Iba1-positive microglia (green) were activated (increased CD68-positive cells (red)) in the presence of cocaine (Additional file 3: Figure S3).

## Discussion

Glia-mediated inflammation underlies several neurodegenerative pathologies including Alzheimer’s disease, Parkinson’s diseases, multiple sclerosis, and HAND. Increased release of pro-inflammatory mediators such as cytokines and chemokines from microglia (brain resident macrophages) and the toxicity of these factors on the neighboring neuronal and astrocytic cells are thought to underlie disease pathogenesis in many of these disorders. Microglia constitute the first line of defense and respond to multiple stimuli leading either to neuronal protection (moderate/controlled reaction) or neurotoxicity (over-activation). Emerging evidence has implicated the role of psychostimulants such as cocaine and methamphetamine in promoting microglial activation leading, in turn, to increased secretion of a plethora of pro-inflammatory cytokines such as IL-1β, IL-6, and TNFα. The signaling pathways involved in cocaine-mediated induction of microglial activation, however, remain elusive and were the focus of this current study.

TLRs belong to a family of pattern recognition receptors (PRR) that are abundantly expressed in various brain cells including but not limited to microglia [45]. These receptors play key roles in regulating and initiating the microglial immune responses. Upon activation by their respective ligands, TLRs (except for TLR3) recruit a number of adapter proteins including the Myd88 to initiate the pro-inflammatory TLR/Myd88/NF-ĸB cascade. In addition to their respective ligands, TLRs have also been shown to be activated by other factors including but not limited to drugs of abuse. As an example, opiates such as morphine can up-regulate the expression of Myd88 in the rat spinal cord, which is likely involved in the development of tolerance to morphine-induced analgesia [46]. Furthermore, morphine exposure has also been shown to modulate the expression of microglial TLRs (TLR2/4), thereby contributing to accelerated neuropathogenesis in a model of human immunodeficiency virus (HIV)-1 infection [47]. Additionally, psychostimulants such as methamphetamine have also been implicated in exacerbating neuroinflammation via activation of TLR9-mediated pathways [48]. Furthermore, cocaine has also been shown to interact with microglial TLR4 to trigger pro-inflammatory signaling, which, in turn, was critical for the neurochemical and behavioral changes induced by cocaine [49]. While these studies shed light on cocaine-mediated activation of microglial via the TLRs, the precise molecular mechanism(s) underlying the interaction between cocaine and TLR4 remain poorly understood.

In our previous findings, we have demonstrated that cocaine activated microglia through ER-dependent stress pathway(s) [50]. Herein, we demonstrate yet another novel mechanism involving the TLR2 pathway by which cocaine exacerbates microglial activation both in vitro and in vivo. We have shown that cocaine not only induced levels of TLR2 protein in a time- and dose-dependent manner, but also activated phosphorylation-dependent signaling of the TLR2 pathway. These findings were further corroborated using the genetic knockdown approach wherein blocking TLR2 resulted in dampening of cocaine-mediated activation of microglia as evidenced by abrogation of released pro-inflammatory mediators such as IL-6, TNFα, and MCP-1. Our previous study demonstrated that sigma-1R/lipid rafts played a critical role in NADPH-mediated ROS generation in cocaine-exposed microglial cells [36]. The current findings further implicate the upstream ROS-ER stress axis in cocaine-mediated induction of TLR2 protein as evidenced by the fact that both the ROS scavenger (PBN) and the NAPDH oxidase inhibitor (APO) abrogated cocaine-mediated induction of TLR2 and subsequent microglial activation. Our findings regarding cocaine-mediated activation of ROS are in agreement with other reports wherein chronic cocaine exposure in rats was shown to enhance cardiac oxidative stress leading, in turn, to cardiac dysfunction [51]. Cocaine exposure was also shown to induce oxidative damage to the skin via the xanthine oxidase and nitric oxide synthase pathways [52]. The oxidative effects of cocaine in this study were also evident in other tissues such as the liver, kidney, and heart [53]. Taken together, ROS appears to be a common mediator in cocaine-induced toxicity in various disease model systems. In line with these reports, our findings also provide evidence that ROS plays a crucial role in cocaine-mediated activation of microglia, thereby suggesting its role in neuroinflammation. Our results also demonstrated that exposure of microglia to cocaine resulted in increased phosphorylation of ER stress sensors, PERK and eIF2α, followed by induction and nuclear translocation of ATF4. This, in turn, resulted in increased binding of ATF4 to the first intron of TLR2. Both pharmacological blocking of microglia with the ER stress inhibitor 4-PBA and genetic silencing of ATF4 ameliorated cocaine-induced effects on microglia. In this study, we have demonstrated that the ROS-ER stress-ATF4 pathway underlies cocaine-mediated effects on microglial activation involving TLR2. Although our findings suggest a role of cocaine in microglial activation, it must be acknowledged that there are controversial reports in literature about the lack of microglial activation following cocaine exposure [54, 55]. The discordant results could, in part, be attributable to concentrations of cocaine and/or other experimental paradigms. As shown in Additional file 4: Figure S4, a single injection of 20 mg/kg cocaine to the mice does not lead to microglial activation in vivo.

The effect of cocaine-mediated microglial activation could have significant ramifications in various pathological conditions such as HAND—often linked and accompanied with substance abuse co-morbidity [56–58]. It is becoming well-recognized that while antiretroviral drugs are the gold standard for HIV care and are effective in suppressing peripheral viremia, the relative inability of these drugs to penetrate the blood-brain barrier, the latency of HIV in the tissues, and the increased life span of individuals on therapy have increased the prevalence of HAND in the post-antiretroviral therapy (ART) era. Adding further complexity is the underlying anxiety and depression that is fairly common among HIV-infected individuals which, in turn, further predisposes the subjects to substance abuse [59].

Various in vitro findings have demonstrated that exposure to cocaine alone or in combination with HIV proteins leads to augmented activation of various CNS cells [34, 35, 60]. Our previous studies have also shed light on the ability of cocaine to potentiate HIV gp120-mediated neuronal and astrocyte apoptosis [35, 61]. Moreover, cocaine has also been demonstrated to induce vascular permeability through the induction of platelet-derived growth factor (PDGF) [62]. Cocaine exposure has also been shown to exacerbate HIV replication in monocyte-derived macrophages and bring the virus out of latency in U1 cells [63]. Cocaine thus acts at various levels on different cell types to contribute to the pro-inflammatory milieu observed during HIV infection. It can thus be classified as a multifactorial agent that mediates its effects on several signaling pathways in HIV-1 infected cells. Not only does the drug promote virus replication in PBMCs, macrophages, microglia, and astrocytes, but it also can modulate glial function and activation [64, 65]. Cocaine causes interactive neurotoxicity with viral proteins such as Tat and gp120, thereby exacerbating neuronal apoptosis. Additionally, cocaine exerts potent effects on microvascular permeability leading to increased influx of virus-infected inflammatory cells in the brain parenchyma [3, 36, 62].

## Conclusions

**Fig. 8:**
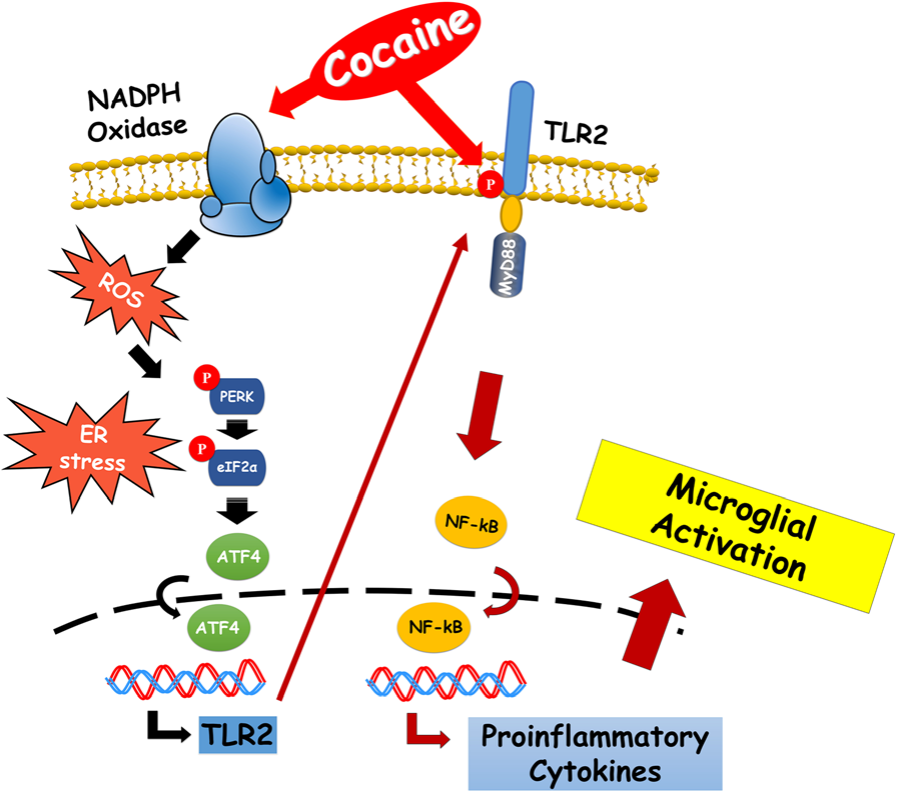
Schematic of signaling pathways involved in cocaine-mediated induction of microglia TLR2 expression and activation. Cocaine induces NAPDH oxidase-mediated accumulation of ROS with downstream activation of the ER-stress PERK/eIF2α/ATF4 pathways which, in turn, leads to ATF4 translocation, with the subsequent induction of TLR2. Additionally, cocaine also activates the phosphorylation-dependent signaling of the TLR2 pathway leading ultimately to microglial activation

In summary, our findings delineate detailed molecular mechanism(s) underlying cocaine-mediated activation of microglial cells via NAPDH oxidase-mediated accumulation of ROS with downstream activation of the ER-stress PERK/eIF2α/ATF4 pathways which, in turn, leads to translocation of ATF4 and subsequent induction of TLR2 protein. Additionally, cocaine also activates the phosphorylation-dependent signaling of the TLR2 pathway. Ultimately, cocaine exposure also results in microglial activation (Fig. 8). Our current findings on the molecular pathway(s) involving the ROS-ER stress-ATF4-TLR2 axis in cocaine-mediated activation of microglia further corroborates the multifactorial role of this drug in exacerbating neuropathogenesis. Therapeutic strategies aimed at blocking the ER stress and/or TLR2 pathway could pave way for dampening the microglial activation in various neurodegenerative disorders with or without cocaine co-morbidity.

## Additional files

Additional file 1: Figure S1.
**Effects of PBN (ROS scavenger) and APO (NADPH oxidase inhibitor) on cocaine-induced ROS formation in BV2 cells.** (A) Green fluorescence was observed using a Zeiss Observer Z1 inverted microscope (Carl Zeiss MicroImaging). It revealed that PBN and APO resulted in significant inhibition of cocaine-mediated induction of ROS production. (JPG 7600 kb) Additional file 2: Figure S2.
**Cocaine-mediated activation of ER stress (PERK/eIF2α/ATF4 pathway) in the BV2 cells.** BV-2 cells were treated with cocaine at the indicated times and whole cell lysates were subjected to western blots to detect the levels of ER stress proteins. Cocaine exposure resulted in time-dependent phosphorylation of PERK and eIF2α proteins and up-regulation level of ATF4 protein. (JPG 1560 kb) Additional file 3: Figure S3.
**Seven days cocaine administration resulted in microglial activation in the striatum of C57BL/6N male mice.** (A) Adult mice, 25–30 g, were administered with cocaine (20 mg/kg/day) by IP injection for 7 days and sacrificed 1 h after the final injection for preparing brain tissue sections. Double immunostained floating tissue samples with Iba1/CD68 (microglia activation marker), which was gelatin mounted, was assayed for co-localization of Iba1/CD68 using the nuance multi spectrum imaging system. It demonstrated that cocaine induced CD68 (red) expression which co-localized with microglia (Iba1, green) in the mouse brain. (JPG 5520 kb) Additional file 4: Figure S4.
**One day cocaine administration had no effect on induction of microglial activation and TLR2 protein levels.** (A) Adult mice, 25–30 g, were administered with one day IP injection of cocaine (20 mg/kg). And striatal homogenates were assessed for levels of CD11b (microglia activation marker) and TLR2. No significant difference of CD11b and TLR2 protein levels between saline and cocaine injection group. (JPG 2890 kb)

## Abbreviations

4-PBA: sodium 4-phenylbutyrate
APO: apocynin
ATF6: activating transcription factor 6
ChIP: chromatin immunoprecipitation
CNS: central nervous system
eIF2α: eukaryotic initiation factor 2α
ER: endoplasmic reticulum
HAND: HIV-associated neurocognitive disorders
HIV: human immunodeficiency virus
Iba1: ionized calcium-binding adapter molecule 1
IRE1: inositol-requiring kinase 1
Myd88: myeloid differentiation protein 88
PBN: phenyl-*N*-t-butyl nitrone
PERK: protein kinase RNA-like endoplasmic reticulum kinase
ROS: reactive oxygen species
si-Con: non-sense siRNA
siRNA: short interfering RNA
TLR2: toll-like receptor-2
UPR: unfolded protein response
