# Supplementary figures and images for: Cocaine-mediated induction of microglial activation involves the ER stress-TLR2 axis
Source: J Neuroinflammation. 2016 Feb 9;13:33. doi: 10.1186/s12974-016-0501-2 (PMC4748483; doi:10.1186/s12974-016-0501-2)

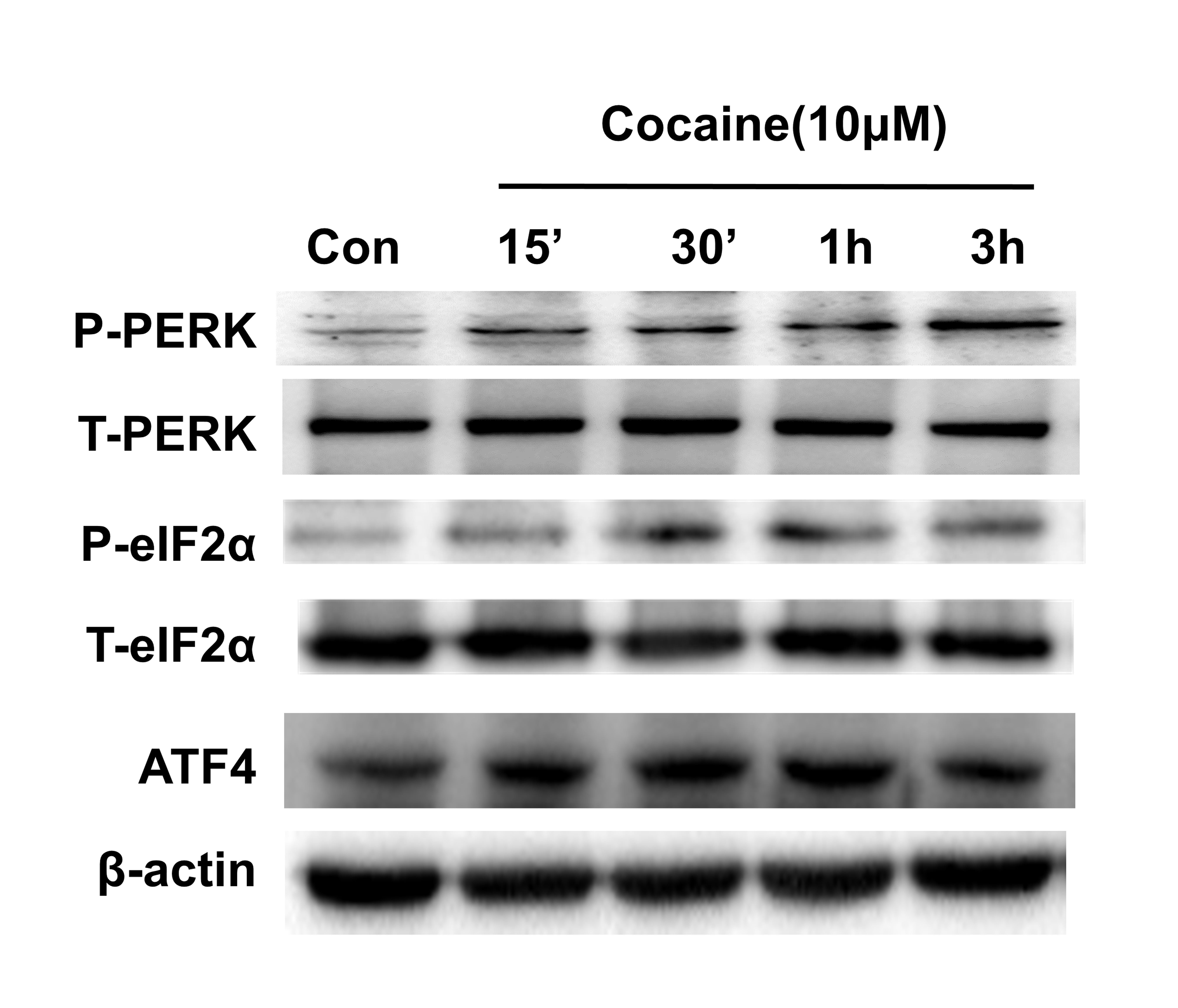

Supplement: Additional file 2: Figure S2. — Cocaine-mediated activation of ER stress (PERK/eIF2α/ATF4 pathway) in the BV2 cells. BV-2 cells were treated with cocaine at the indicated times and whole cell lysates were subjected to western blots to detect the levels of ER stress proteins. Cocaine exposure resulted in time-dependent phosphorylation of PERK and eIF2α proteins and up-regulation level of ATF4 protein. (JPG 1560 kb) [file 12974_2016_501_MOESM2_ESM.jpg]
